# Protocol using *ex vivo* mouse brain slice culture mimicking *in vivo* conditions to study tumor growth and cell motility of glioblastoma cells

**DOI:** 10.1016/j.xpro.2024.103401

**Published:** 2024-10-19

**Authors:** Laura Neises, Catherine Delbrouck, Anne Schuster, Mahsa Rezaipour, Kim Eiden, Anais Oudin, Carina Fabian, Simone P. Niclou, Anna Golebiewska, Johannes Meiser

**Affiliations:** 1 Cancer Metabolism Group, Department of Cancer Research, Luxembourg Institute of Health, L-1210 Luxembourg, Luxembourg; 2 Department of Life Sciences and Medicine, Faculty of Science, Technology and Medicine (FSTM), University of Luxembourg, L-4367 Belvaux, Luxembourg; 3 NORLUX Neuro-Oncology Laboratory, Department of Cancer Research, Luxembourg Institute of Health, L-1210 Luxembourg, Luxembourg

**Keywords:** cell culture, cancer, tissue engineering

## Abstract

Herein, we present an *ex vivo* approach to study glioblastoma (GBM) cell motility in viable mouse brain slice cultures, closely mimicking *in vivo* features. We detail the preparation and culturing of mouse brain slices followed by tumor cell injection, allowing for the analysis of different aspects of the cellular migration and invasion process. Our assay facilitates testing diverse perturbations including genetic modifications and treatments in a physiological context. Thus, the protocol provides a compromise between *in vitro* assays and *in vivo* models.

For complete details on the use and execution of this protocol, please refer to Delbrouck et al.^1^ and Schuster et al.^2^

## Highlights

• Mouse brain slice cultures allow for functional tumor assays *ex vivo*
• Tumor cells can be injected into the brain tissue and integrate therein
• Motility of integrated tumor cells can be studied in highly physiological brain environment
• Functional assessment of cell motility combined with genetic perturbations or treatments

## Abstract (editor highlights)

Publisher’s note: Undertaking any experimental protocol requires adherence to local institutional guidelines for laboratory safety and ethics.

## Graphical abstract

**Figure:**
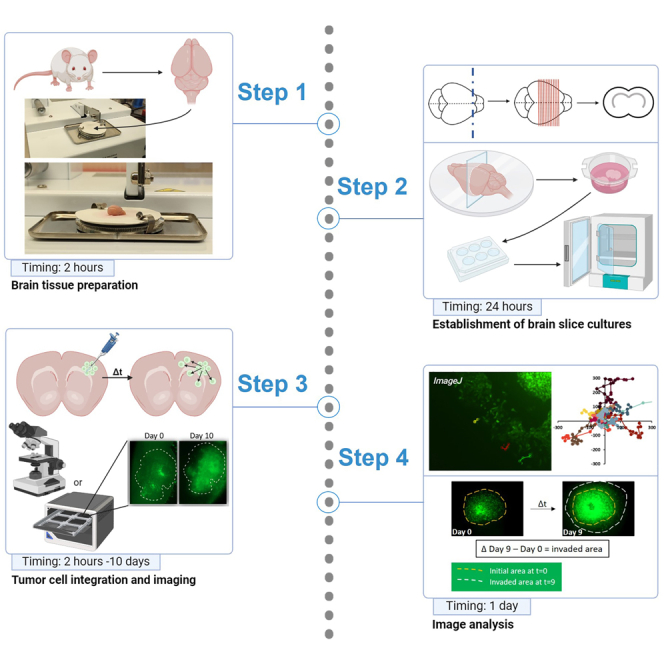

## Before you begin

The protocol describes the experimental steps to study tumor growth and motility of fluorescently labeled tumor cells in mouse brain tissue. We have optimized the protocol with use of patient-derived GBM cultures. Our initial work was based on LN-229 GBM cells that were genetically modified with either a knockdown of *MTHFD1L* (LN-229 sh*MTHFD1L*^1^) and their specific non-targeting control (LN-229 sh*SCR*^1^) and the respective parental line LN-229 wild type (wt). Genetic engineering was designed to integrate stable expression of Green Fluorescent Protein (GFP) to monitor gene knockdown status and cell tracing. In another study, we applied a similar strategy to GBM stem-like cultures, where tumor cells were stably labeled with GFP (control, sh*SCR*, sh*ZFAND3* and *ZFAND3*-overexpressing conditions^1^^,^^2^). The protocol requires access to fresh viable mouse brain tissue. To visualize integration and perturbation of tumor cells inside the brain structures over time, fluorescent labeling is necessary. The fluorescence-based labeling may be achieved via stable transfection as well as fluorescent labeling pre-injection.^3^ The experimental protocol can be performed for up to seven to ten days. If available, a live cell imaging system, such as the IncuCyte Live Cell Analysis Instrument, may be of advantage for quantification and analysis.

### Institutional permissions

In our work we have applied patient-derived adherent GBM cell lines and stem-like cultures, with stable expression of GFP.^1^^,^^2^ All models were derived from patient tissue following informed consent of the patients. Animals were housed in a specific pathogen free (SPF) animal facility, under controlled environment (temperature, humidity and light) with free access to water and food *ad libitum*. Mice euthanasia processes strictly followed the European Directive 2010/63/EU. The euthanasia for organ sampling are done by competent persons and in an authorized user/breeder establishment.

**Figure 1:**
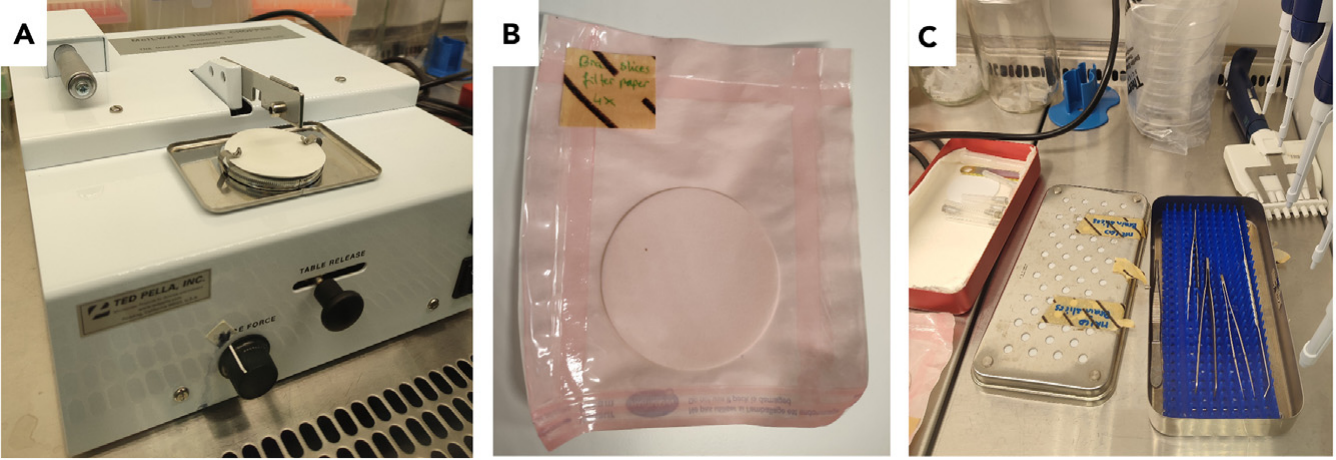
Overview of crucial material needed for preparation of brain tissue slices This includes (A) McIlwain Tissue chopper, (B) sterilized circular Whatman paper and (C) box of sterilized tweezers and spatulas.

### Preparation of cells, culture medium and other material

#### On the day prior the start of the experiment

**Timing: 60 min**

1. Defrost tumor cells of interest in due time to obtain sufficient numbers of viable cells for injection 24 h after isolation of the mouse brains.

***Note:*** Ensure that the cells can recover from the defrosting for one to two days and then be cultured for at least two passages before using them for injection into the mouse brain slices.

***Note:*** At this stage, tumor cells should ideally be mycoplasma-free and >90% viable.

**CRITICAL:** Ensure that the cells you want to inject into the mouse brain slices durably express a fluorescent marker or plan an additional staining prior the injection (see limitations).

2. Order animals or mouse brain tissue from the animal facility following institutional procedures.

***Note:*** Fresh mouse brains are required for the protocol. We regularly apply brains extracted from male and female mice aged between 12–20 weeks. We would like to highlight that we used mice of a huge range of age for our technique since we wanted to use surplus or even unused animals. Consequently, we did not produce mice specifically for the purpose of the protocol. Since we regularly perform xenografting of human tumor cells in immunodeficient NOD. Cg-Prkdc^scid^ Il2rg^tm1WjI^/SzJ (NSG) and nude male and female mice aged between 8–16 weeks, we applied the same strains to the *ex vivo* brain slice cultures.

***Note:*** To retain similar conditions as in *in vivo* experiments, we recommend to use immunodeficient mouse stains for injecting human tumor cells. However, since T cells are rare in healthy brain parenchyma, we do not expect host versus graft effects *ex vivo* in immunocompetent mouse strains. Parenchymal microglia and non-parenchymal border-associated macrophages, which represent innate immune cells in the healthy brain, do not cause host versus graft disease.

***Note:*** Mice should be euthanized by competent personnel following the national and EU regulation and according to institutional permissions, cervical dislocation (if needed with CO_2_ overdose) can be applied. For cervical dislocation, restrain the mouse on grid, place two fingers behind the base of the skull to firmly block the head and pull back strongly on the tail to induce a rapid separation between skull and the high cervical vertebrae.

**CRITICAL:** To ensure an efficient cutting process resulting in high quality tissue slices, we advise to process a maximum of four mouse brains at the same time. The prolonged storage of the brains extracted from the skull adversely impacts the tissue cutting procedure and subsequently quality of the brain slices.

3. Prepare for the setup of a tissue chopper, ideally the McIlwain Tissue chopper (Figure 1A), and compare the machine with the instrument manual to confirm that all essential machine parts are available. To ensure usage of a clean and sterile cutting blade, place at least two blades in a small bucket filled with 70% of Ethanol (EtOH) and store at +4°C.
4. Prepare all required solutions and culture media under sterile conditions. Once prepared, store the solutions at +4°C in a closed standard sterile bottle.

***Note:*** Standard cell culture medium, DMEM has been used for our experiments described in this protocol. This medium can be replaced by other standard cell culture media, depending on the cell type being used. See in the materials and equipment for exact formulation of the cutting solution and the brain slice medium.

5. Cut the Whatman filter paper in circles of an average diameter of 60 mm, covering the size of the cutting disk.

***Note:*** Use a fresh lid from a 60 × 15 mm^2^ petri dish as a template. Plan at least one filter paper per mouse brain. Prepare some filter papers in excess.

6. Sterilize the circular Whatman filter papers, two pairs of metallic tweezers (Figure 1B) and a flat spatula (Figure 1C).

***Note:*** Sterilize material using dry sterilization in an autoclave at 121°C at 205 kPa for 30–60 min or equivalent.

7. For enhanced shelf-life, aliquot the extracellular matrix (ECM) (Sigma-Aldrich, Cat. no. E1270) stock solution into 10–20 μL aliquots under sterile conditions and store at −20°C. Avoid freeze-thaw cycles.

**CRITICAL:** Defrosting of ECM stock solution at +4°C may require several hours depending on the total volume. Aliquoting procedure requires +4°C.

## Key resources table

**Table:** 

REAGENT or RESOURCE	SOURCE	IDENTIFIER
**Chemicals, peptides, and recombinant proteins**		

ECM Gel from Engelbreth-Holm-Swarm murine sarcoma	Sigma-Aldrich	E1270
BIT-100	Provitro	2043100
Heparin	STEMCELL Technologies	07980
L-glutamine	Thermo Fisher Scientific	25030081
Glucose	Merck	G8769
GlutaMAX	Thermo Fisher Scientific	35050061
DMEM/F12	Thermo Fisher Scientific	11320033
DMEM	Thermo Fisher Scientific	A14430
Pen/Strep	Westburg	LO DE17-602E
HEPES	Thermo Fisher Scientific	15630080
70% EtOH	VWR	85825.360
Hibernate A-medium	Thermo Fisher Scientific	A1247501

**Experimental models: Cell lines**		

Human: LN229	ATCC	CRL-2611

**Experimental models: Organisms/strains**		

Mouse: NOD.Cg-Prkdc ^SCID^ Il2rg ^tm1Wjl^ /Sz, 8–20 weeks, male and female	J Charles River, France	RRID:IMSR_JAX:005557

**Software and algorithms**		

ImageJ	Version 1.52 a	https://imagej.net/ij/index.html
Live-Cell Imaging & Analysis Software	Sartorius	https://www.sartorius.com/en/products/live-cell-imaging-analysis/live-cell-analysis-software

**Other**		

**Consumables**		

razor blades (blade size 13 mm)	DM	966935
Whatman filter paper	Merck	WHA1001055
Transwell chambers with the pore size of 1 μm	Greiner Bio-One	657610
Glue stick	Pritt	199986

**Machines**		

Tissue chopper	McIlwain	https://www.manualslib.com/manual/1298233/Mcilwain-Tissue-Chopper.html
IncuCyte S3 Live-Cell Analysis System	Sartorius	4647

## Materials and equipment

**Table:** Brain slice medium

Reagent	Final concentration	Amount
Hibernate A	100%	395 mL
BIT-100	20%	100 mL
Pen/Strep	100 U/mL	5 mL
**Total**	**N/A**	**500 mL**

• Store in a closed standard sterile bottle at +4°C for max. 1 month.

**Table:** Modified brain slice medium

Reagent	Final concentration	Amount
Hibernate A	50%	194.3 mL
DMEM-F12	50%	194.3 mL
BIT-100	20%	100 mL
L-glutamine	2 mM	5 mL
Pen/Strep	100 U/mL	5 mL
Heparin	1 U/mL	1.39 mL
**Total**	**N/A**	**500 mL**

• Store in a closed standard sterile bottle at +4°C for max. 1 month.

**Table:** Cutting solution

Reagent	Final concentration	Amount
DMEM	50%	478.3 mL
GlutaMAX	0.1%	0.5 mL
Glucose	17.4 mM	3.7 mL
HEPES	25 mM	12.5 mL
Pen/Strep	100 U/mL	5 mL
**Total**	**N/A**	**500 mL**

• Store in a closed standard sterile bottle at +4°C for max. 1 month.

## Step-by-step method details

### Brain tissue slice preparation

#### On the day prior to the cell injection into the brain slice

**Timing: 1.5 h**

**Timing: 60 min (for step 1)**

**Timing: 5 min (for step 2)**

**Timing: 25 min (for step 3)**

This major step divided into three parts describes the precise setup of the tissue chopper, the isolation of the brain from the mouse and the cutting of the freshly isolated mouse brain into tissue slices.

**Figure 2:**
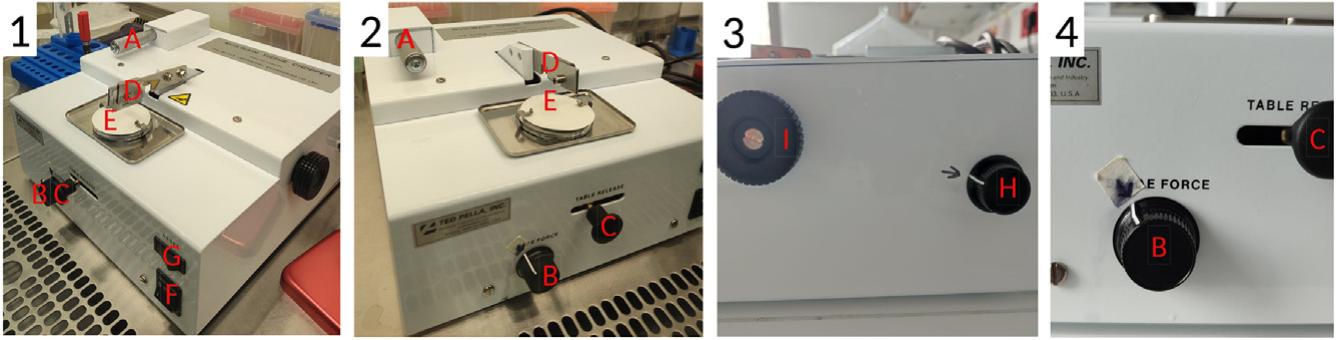
Essential instrument parts of the McIlwain tissue chopper shown placed in the BSCII Essential parts of the machine are shown as: (A) slice thickness adjustment micrometer, (B) blade force control knob, (C) table release knob, (D) blade holder, (E) cutting disk with filter paper placed on top, (F) On/Off switch, (G) reset switch, (H) speed control knob, (I) manual operating knob. (3) Close-up view on precise orientation of speed control knob (item H). (4) Close-up view on precise orientation of blade force control knob (item B).

**Figure 3:**
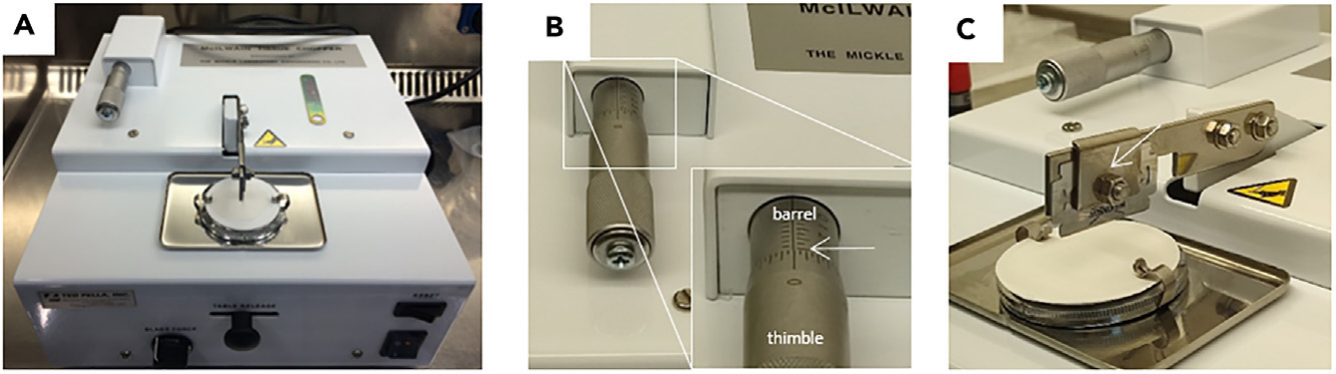
Detailed view on McIlwain tissue chopper (A) Overview of machine with more precise view on (B) slice thickness adjustment micrometer composed of barrel and thimble to be set to “4" (white arrow) corresponding to a cutting distance of 400 μm and (C) cutting blade fixed to the blade holder (white arrow).

**Figure 4:**
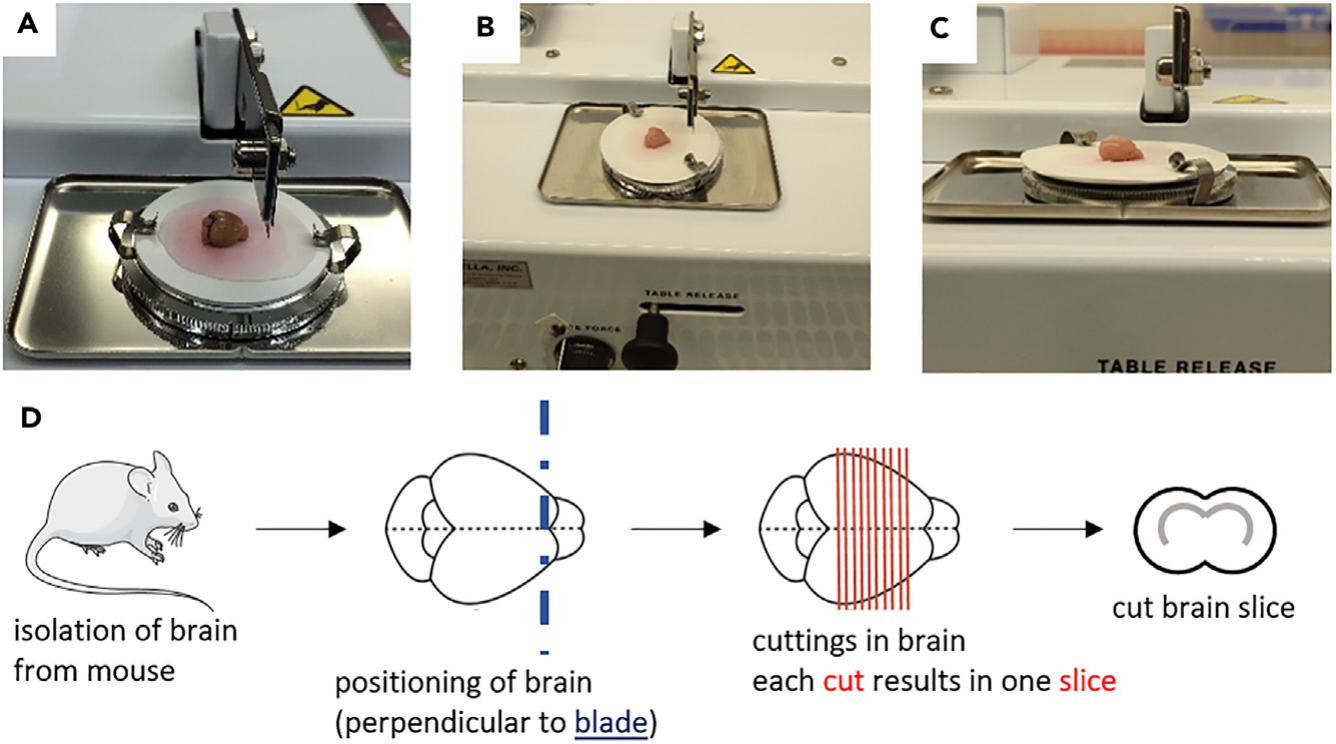
Mouse brain placed on the cutting disk under the blade (A) Close view from top, (B) zoom out view from top and (C) view from the side. (D) Schematic overview of mouse brain cutting process, essential parts are highlighted in colors.

**Figure 5:**
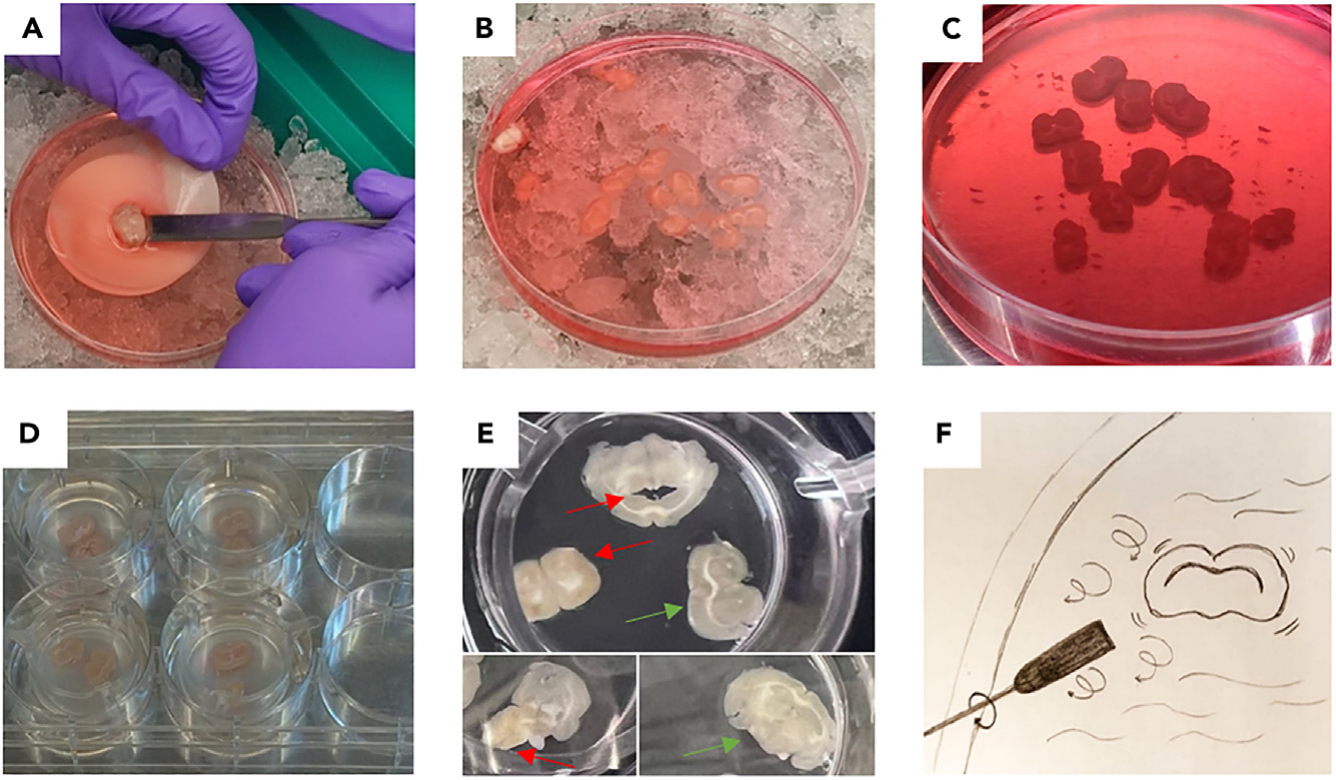
Recovery of the cut brain and separation of the mouse brain slices (A) Transfer of the cut brain into the cutting solution in the Petri dish. (B) Separated mouse brain slices in cutting solution stored on ice and (C) Zoom in. (D) View on the brain slices in the transwell chambers from the side. (E) Close up view on freshly cut brain slices: Upper image part shows two slices (indicated by a red arrow) that were excluded from further experimentation due to two layers of brain slice attaching together (bottom left) and disrupted tissue (up); the intact slice indicated by the green arrow had been used for cell injection. Lower part left shows a slice where half of the tissue remains folded and thus the slice was excluded from further experimentation. Lower part right shows an intact slice. (F) Sketch illustrating the creating of soft turbulences in the ice-cold cutting solution using the spatula to facilitate lifting and thus transfer of such onto the transwell chamber membrane.

1. Setup of the tissue chopper.
  ***Note:*** To maintain sterile conditions, all required material needs to be placed in a biosafety cabinet class II (BSCII).
  ***Note:*** The following steps describing the instrumental setup of the tissue chopper need to be done once before starting with the tissue cutting process.
  a. Place the tissue chopper (McIlwain, Figure 3A), two cooling boxes filled with ice, the pre-cooled cutting solution (placed in a box of ice), a petri dish (diameter of 100 mm^2^), the tweezers, the spatula as well as the glue stick (Pritt, Cat. no. 199986) in the BSCII.
  b. Pre-warm brain slice medium directly in the bottle to 37°C.
  c. Properly clean the surface of the tissue chopper by wiping off the instrument with 70% of Ethanol.
  d. Ensure correct installation of the tissue chopper. Apply the following settings:
    i. Set up the control knobs for speed (Figure 2, part 1 and 3, item H) and blade force (Figure 2, part 2 and 4, item B) to a user-defined level. The optimal cutting speed and blade force were empirically tested and determined as follows under CRITICAL.
      **CRITICAL:** The optimal cutting speed is required to eliminate the risk of the brain slices attaching to the razor blade (speed too low) or to each other (speed too high). The blade force should be sufficientlystrong to cut the filter paper underneath the mouse brain. In case of partial paper cut, increase the blade force. For more visual insight on the cutting procedure, watch Methods videos S1, S2, S3, and S4.
      ***Note:*** In our experience, the tissue slice thickness (i.e. the cutting distance) of 400 μm is the most optimal for high brain tissue viability during seven to ten days in culture. Slice thickness can be adjusted to the experimental needs and live/dead assays may be useful to assess the ideal slice thickness.
      ***Note:*** Live/dead discrimination can be applied in the viable tissue with the viable cell membrane impermeable dyes such as DAPI (4′,6-diamidino-2-phenylindole) or PI (propidium iodide). Optionally, brain slices can be embedded in paraffin and use for IHC-based visualization of tissue quality.
    ii. Turn the thimble of the slice thickness adjustment micrometer (Figure 2, item A) clockwise until its outer rim reaches the marking line “4” on the barrel, as shown by the white arrow in Figure 3B.
    iii. Adjust the marking on the thimble to “0” to be in line with the lining on the barrel.
      ***Note:*** The following steps describing the preparation of some accessory instrumental material need to be repeated for every fresh mouse brain that will be cut.
  e. Prepare a sterile 6-well tissue culture plate, at least six transwell chambers with the pore size of 1 μm (or alternatively 3 μm pore size) and pipettes in the range of 1000 μL–10 μL ready-to-use.
  f. Use the pair of tweezers to transfer a sterile round-cut Whatman filter paper on top of the tissue chopper.
    i. Carefully apply a thin layer of glue on one side of the paper, covering the entire surface.
      ***Note:*** Avoid unnecessary touching of the filter paper with your gloves to maintain sterility of the filter paper.
    ii. Attach it with the adhesive surface directly on the cutting disk (Figure 3C), so that the filter paper adheres to the cutting disk.
      ***Note:*** The filter paper serves as a cushioned support on the cutting disk for the mouse brain to be placed for the cutting. Glue is needed to adhere the filter paper to the cutting disk.
      **CRITICAL:** Apply the glue indicated in the material section. Other adhesive material may interfere with the culturing of the brain slices.
  g. Carefully place the cutting disk on top of the cutting table on the tissue chopper by tightly closing the two clips.
  h. By using the pair of tweezers, take out the blade of the 70% EtOH container and gently dry it on a piece of paper towel.
  i. Meanwhile, unscrew the blade holder and carefully insert the blade (Figure 3C).
  j. Fix the blade horizontally in line with the blade holder and then tightly screw the holder.
2. Isolation of the brain from the mouse.
  ***Note:*** Work fast but precise for the following steps. Always ensure proper cooling of the tissue and careful handling of such.
  ***Note:*** If you decide to perfuse the brain, prepare everything needed to do so at this stage of the experiment. Continue after with the next step of the protocol.
  a. Fill 5 mL of cold cutting solution in a 15 mL Falcon tube and place on ice. Isolate the brain from the mouse as described in the following steps.
  b. After death confirmation of the animal, decapitate the mouse head at the base of the skull.
  c. Cut the scalp from midline until the middle of the eyes.
  d. Turn over the scalp to expose properly the skull and firmly fix the brain with the scalp.
  e. Insert the delicate bone trimmer in the occipital condyle, stay in ventral part as much as possible and cut on right and left part to make extra space, to easily remove the occipital bone and intraparietal bone. At this stage, the brain stem and the cerebellum are exposed.
  f. Insert the bone trimmer on each side along of the parietal bone and cut. Insert the trimmer along of the midline (pay attention to not insert it into the brain).
  g. Cut with the premaxilla and nasal bone perpendicularly. Remove carefully the parietal and frontal bones of each side.
  h. Extract the brain with a spoon, pay attention to the meninges and the chiasma optic.
  i. Carefully transfer the brain into the 15 mL Falcon tube. Use one Falcon tube per brain.
3. Cutting of the mouse brain.
  **CRITICAL:** Proceed with the cutting of the brain as soon as possible. To ensure a smooth cutting of the freshly isolated brains, do not store them longer than four hours before cutting and ensure continuous storage at +4°C. Otherwise, difficulties during the cutting process may occur, including adhesion of the tissue to the blade or even sticking of the tissue slices to each other.
  ***Note:*** The longer the brains are kept unprocessed, the greater is the quality loss of the cut brain slices. Brains that were isolated more than four hours prior to starting the cutting process may no longer be usable to cut the tissue into slices.
  a. Place the dissected mouse brain in the Falcon tube stored on ice under the BSCII.
  b. Place the petri dish on ice and fill it with 10 mL of the ice-cold cutting solution.
  c. Transfer the mouse brain into this petri dish.
  d. Immediately after, use the tweezers and carefully transfer the brain onto the filter paper on top of the cutting disk, with the olfactory bulb directly under the blade (Figures 4A–4C).
    **CRITICAL:** Do not over-dry the brain; ensure tissue moistening by adding two-three drops of ice-cold cutting solution on top of the brain. Too much cutting solution may lead to wet brain tissue adhering to the blade during cutting. Once you placed the brain on the filter paper, do not move it anymore.
    ***Note:*** Adjust the position of the cutting disk by horizontally moving the table release lever (Figure 2, item C). The positioning of the blade can be adjusted using the blade holder and its screw (Figure 3C).
  e. Ensure that the blade is in perpendicular position to the brain (Figure 4D), you can still adjust the position by turning the cutting table.
    ***Note:*** For the scope of this protocol, we only cut the brain in coronal orientation. This cutting orientation comes with the advantage of easy handling of the brain to bring it in the correct position for the cutting process. Moreover, for downstream analysis of tissue from *in vivo* experiments, we cut the respective tissue in the same fashion. We thus did not test cutting of the brain in axial or sagittal orientation.
    **CRITICAL:** Proceed to cutting fast, since the increasing temperature of the mouse brain tissue will strongly affect the cutting efficiency.
  f. Once tissue position is correctly adjusted, flip the On/Off switch to “On” and start cutting the brain into 400 μm thin slices. Visually supervise the cutting process and hold it if necessary by flipping the reset switch (Figure 2, item G).
    ***Note:*** See Methods videos S1, S2, and S3 for an efficient cutting process, see Methods video S4 for a compromised cutting procedure leading to inadequate slice quality after half of the brain was cut (Methods videos S1, S2, S3, and S4).
    **CRITICAL:** If brain slices start attaching to the blade, immediately stop the cutting process to release the slice from the blade. This is very crucial as otherwise, the attached tissue slice will negatively affect the quality of your further cutting, see Troubleshooting 1.
    ***Note:*** Generally speaking, one can state that the longer the brain stays on top of the cutting disk, so the longer the cutting process lasts, the more the temperature increases in the tissue. Cold tissue is very rigid and can be cut without great difficulty. The warmer the tissue becomes, the sticker it gets. Thus, it can also be challenging to restart the cutting after having paused the process (Troubleshooting 2).
  g. Cut the brain entirely to reach approximately ten brain slices.
    ***Note:*** Only brain slices with visible corpus callosum (white matter) can be used for injection of the cells (Figure 5E). This tissue structure has been defined in terms of technical reasons to meet the same criteria as those set for our *in vivo* experiments. Likewise, one thereby ensures that all brain tissue slices used for cell injection retain the same morphological structures and can thus be compared to each other.
    ***Note:*** We recommend to use ten brain slices as technical replicates per experimental condition for cell injection in step 11. The slices do not necessarily have to come from the same mouse. In our experiments, we processed several mouse brains subsequently and afterwards mixed the conditions over the different mice.
    **CRITICAL:** Anticipate approximately 10–30% loss of brain slices during the procedure, and thus prepare excess tissue slices for the injection of the tumor cells. You may lose some tissue slices during the cutting procedure due to unequal cutting, where after some slices may end up with disrupted tissue parts (see Figure 5E, indicated with red arrows). The percentage of loss strongly depends on the quality (the time they stay uncut after isolation from the animal) of the mouse brains and the experience in handling tissue during the cutting process.
  h. Unlock the clips on the cutting disk to loosen the filter paper and the cutting disk.
  i. Carefully transfer the cutting disk together with the cut brain on the filter paper or the brain solely into the petri dish filled with cutting solution (Figure 5A).
  j. Carefully disassemble the cut filter paper stripes. It will allow to gently separate the single brain slices (Figures 5B and 5C). See Troubleshooting 3 for other means to disassemble the cut brain into single tissue slices.
    ***Note:*** Alternatively, using tweezers, carefully grab the pack of brains slices – they still tightly stick together at this point- and transfer the brain into the cutting solution. Be aware of the increasing risk of damaging the tissue once touching it with the tweezers.
    **CRITICAL:** Avoid damaging the thin tissue slices. Select the slices that still maintain all essential brain structures needed for the respective type of experiment, in our case we selected only those slices with the corpus callosum clearly visible (Figure 5E). Ensure that the tissue slice is still intact as a whole.
  k. Place transwell chambers into the wells of a 6-well tissue culture plate by hanging them each inside one single well.
    ***Note:*** In our hands, maximum two brain slices can be placed per chamber for optimal follow-up experimental procedures.
  l. Using the flat spatula, carefully lift one brain slice after the other and transfer them to the transwell chambers. To place brain slices correctly in the transwell, tilt the spatula to one side, allowing the brain to slowly slip onto the membrane of the transwell chamber. If possible, place both brain slices in the same direction, for ease of orientation in subsequent steps, especially during the cell injection.
    ***Note:*** Creating soft turbulences in the cutting solution with the spatula (Figure 5F) may help lifting the brain slice onto the spatula.
    **CRITICAL:** Verify that the brain slice is placed entirely flat on the membrane; any folding of the slice will impede further experiments (Figure 5E). Avoid to no longer touch the slice with the spatula since once the slice is being put on the membrane without any liquid present, it is very fragile. Rather try to unfold it by softly flushing it with some brain slice medium.
    ***Note:*** The two slices placed in the same chamber should not touch each other (Figures 5D and 5E).
  m. Using a pipette, gently remove the cutting solution, which may have accumulated on top of the transwell membrane during the transfer of the brain slices.
  n. Transfer 1000 μL of the pre-warmed brain slice medium into the well of the 6 well plate, right below the transwell chamber.
    ***Note:*** As the chamber is hanging quite deeply in the well, the brain slices will be in direct contact with the brain slice medium. They will not be fully submerged into the medium, as the air liquid interface- which is the standard technique for culture of brain slices- is still maintained. The bottom of the transwell chamber membrane is in contact with the culture medium, allowing nutrients and gases to diffuse upwards. The upper surface of the brain slices is exposed to the air, facilitating better oxygenation.
  o. In order to prevent evaporation of the medium, fill the space between the wells with 10 mL of sterile H_2_O.
  p. Close the plate with its lid and incubate for 18–24 h at 37°C, 5% CO_2_ in a humidified classical incubator.
    ***Note:*** If you continue the cutting process with another brain, use a fresh sterile blade and a fresh piece of sterile filter paper.

Methods video S1. Smooth cutting process of mouse brain into tissue slices using the tissue chopper device Cut tissue slices remain attached together within the set of cut brain slices from the mouse brain. Relevant for cutting of mouse brain tissue slice into slices, related to step 3.

Methods video S2. Moderate smooth cutting process of mouse brain tissue into slices that can still be separated from each other Cut tissue slices tend to adhere to the razor blade and thus to lose their position within the set of cut mouse brain slices. Relevant for cutting of mouse brain tissue slice into slices, related to step 3.

Methods video S3. Moderate smooth cutting process of mouse brain tissue into slices that can still be separated from each other Cut tissue slices tend to adhere to the razor blade and thus to lose their position within the set of cut mouse brain slices. Mouse brain tissue seems to lose stiffness and becomes a little gelatinous. Relevant for cutting of mouse brain tissue slice into slices, related to step 3.

Methods video S4. The cutting process of the mouse brain starts smoothly but loses quality after half of the brain being cut There, the tissue is losing its stiffness and thus slices are folding up in the cutting region. Thus these cut slices will be randomly cut again and can thus not be used for further experiments. Starts good but last slices not usable. Relevant for cutting of mouse brain tissue slice into slices, related to step 3.

Methods video S5. Time course velocity tracking of green fluorescent cells in queued microscopy images using ImageJ Selected cells were individually colored plus a number (1–6) was given for more differentiated analysis. Relevant for analysis of tumor cell velocity, related to 48.

Methods video S6. Time course velocity tracking of green fluorescent cells in queued microscopy images using ImageJ Images were taken using the brightfield view. Selected cells were individually colored plus a number (1–15) was given for more differentiated analysis. Relevant for analysis of tumor cell velocity, related to 48.

### Preparation of ECM-tumor cell suspension

**Timing: 50 min**

This major step accomplishes the preparation of the ECM-cell suspension for injection of the respective tumor cells into the mouse brain slice. We recommend to prepare sufficient cells needed for ten brain slices as technical replicates per experimental condition, with the aim of injecting exactly 1 μL of cell suspension, containing around 2 × 10^4^–5 × 10^4^ cells, per brain slice.

4. Place a box of sterile pipette tips (1–10 μL) at +4°C. Defrost ECM aliquot(s) at +4°C prior the experiment.

***Note:*** Per injection, you will need 1 μL of liquid ECM. Use cold pipette tips for injection of the cells into the brain slices (in step 11) to ensure that the ECM remains liquid prior integration into the tissue. The ECM remains in a liquid state at −20°C, and solidifies as soon as temperature reaches temperatures greater than +4°C.

5. Passage the cells following the dissociation protocol applied regularly per specific tumor cell culture. Count tumor cells to assess their concentration and viability. Aim at cell viability greater than 90%.

***Note:*** The standard protocol is designed for injection of single cells. 3D structures such as spheroids and organoids can also be injected. Feasibility may depend on the size of the 3D cultures and should be tested empirically. Specific testing of Hamilton syringes, plastic tips or glass pipetted is advised.^4^ ECM can be avoided, though an additional caution is necessary to retain tumor cells inside the brain slice structure.

**CRITICAL:** It is crucial to inject the same number of viable cells per condition that will be compared (Troubleshooting 4).

**CRITICAL:** 2 × 10^4^–5 × 10^4^ cells are recommended per injection into one tissue slice. The exact cell number needs to be established for each cell culture depending on the inherent proliferative and invasive properties of the cells, i.e. highly invasive or highly proliferative cells may require fewer cells.

***Note:*** In our case, we empirically determined to inject 5 × 10^4^ cells per brain slice for LN-229 cells. Injection of too few cells into the brain slice may result in tumor cells not properly growing into the tissue due to missing cell-cell contacts. A very high cell number (> 5 × 10^5^ cells) may be difficult to resuspend in 1 μL and injection of such a dense cell suspension can result in improper implantation.

6. Prepare one cell suspension for all technical replicates. Take into account dead volumes during pipetting and prepare excess of single cell solution. e.g., for 20 injections, transfer 1.1 × 10^6^ LN-299 cells in a sterile 1.5 mL Eppendorf tube (for 5 × 10^4^ cells per brain slice).
7. Centrifuge the tubes containing single cells for 3 min at 300 × *g* at 22°C. Discard the supernatant as completely as possible. Ideally, aspirate the remaining supernatant using a 10–200 μL pipette.

**CRITICAL:** All material applied in the follow-up steps should be pre-cooled, ECM should be defrosted at +4°C. Work fast but precise in the next steps and ensure proper cooling of the ECM.

***Note:*** Verify whether the pure ECM is well homogenized, resuspend on ice without creating air bubbles.

8. Resuspend the prepared cell pellet in pure ice-cold ECM to reach 1 μL per desired cell number per one brain slice. e.g., for planned 20 injections, carefully resuspend the cell pellet in 20 μL of ECM on ice. Keep the mixture on ice until the injection.

### Injection of cells into the mouse brain slice

**Timing: 20 min per injection**

In this step, you will inject the tumor cells of interest in the mouse brain slice using the beforehand prepared solution of cells mixed with liquid ECM.

9. Take out the 6-well cell culture plate harboring the transwell chambers with the mouse brain slices from the incubator and place it on a dark surface.

***Note:*** For better visualization of the brain slice structures by eye, we utilized a black mouse pad wiped with 70% EtOH and tissue paper on the surface and placed it underneath the tissue culture plate.

***Note:*** Intactness of the tissue is crucial for successful implantation of the cells. As described by Eisemann et al.,^4^ do neither dehydrate nor excessively immerse the tissue on brain slice media during punching the tissue with the pipette tips (step 11). In our hands, incubating the freshly cut brain slices in 1000 μL of brain slice medium (step n) for 24 h worked well for subsequent injection of the cells.

***Note:*** However, if necrosis or any other degeneration of the tissue can be observed after 24 h of incubation, the respective mouse brain slice cannot be used for subsequent cell injection. We advise to exclude this slice from the experiment.

**Figure 6:**
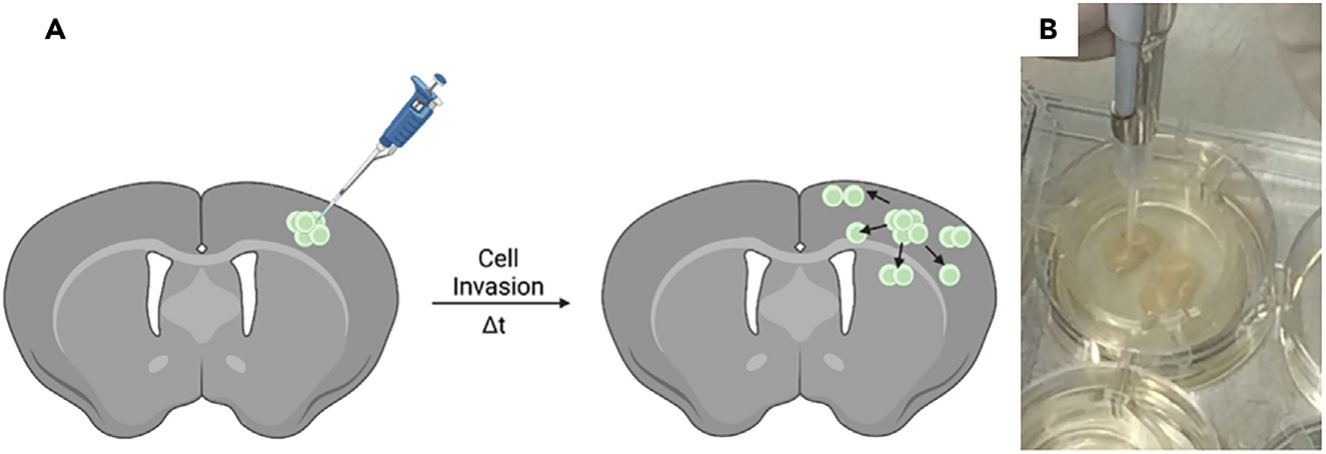
Injection of cells into the mouse brain tissue slice (A) Schematic of injection of fluorescently labeled cells into the mouse brain slice (left) and monitoring of cell invasion into the brain tissue over time (right). Figure created with BioRender.com. (B) Picture taken during injection of cells into a mouse brain slice placed in a transwell chamber.

10. Using a 1–10 μL pipette (and the pre-cooled pipette tips, see step 4), aspirate 1 μL of the ECM-cell mixture. Ensure that the ECM-cell mixture is well homogenized before finally taking of the 1 μL volume needed for cell injection.
11. With the cone of the pipette tip, punch a small hole in the brain tissue, in the cortex, above the corpus callosum (Figures 6A and 6B) and inject the 1 μL while slowly releasing the pipette tip from the mouse brain tissue.

**CRITICAL:** Ensure that the pipette tip is inserted to a depth that reaches the target area without penetrating too deeply. During the injection you will feel slight resistance, indicating that the pipette tip reached inside the tissue. If you do not feel this resistance, it means you are injecting at the tissue surface, which may result in many single cells being left on top of the tissue.

***Note:*** If the pipette tip punctures through the brain slice, it can cause excessive damage and affect the accuracy of the injection. Monitor the resistance felt during insertion. A sudden loss of resistance may indicate that the pipette tip has punctured through the tissue.

***Note:*** Try to punch a hole as small as possible into the tissue, to be just about to smoothly inject the cells into the tissue without damaging the tissue. Tissue slices with bigger punching holes can still be used to downstream analysis, as long as the tissue does not start to degenerate from this step onwards.

***Note:*** It is essential to release the pipette tip from the mouse tissue while injecting the cell suspension, to ensure that the cells remain in the tissue.

***Note:*** Due to pre-existing procedures for potential subsequent *in vivo* experiments, we limited the injection of cells to the cortex and did not expand it to other parts of the brain. Importantly, you need to keep in mind that behavior of invasion may differ in between parts of the brain where you inject the cells due to different microenvironments inside the mouse brain.

12. Immediately after having injected the ECM-cell mixture into a single brain slice, verify whether the cells were injected into the mouse brain tissue by using a fluorescent microscope.

***Note:*** If encountering difficulties to directly inject the cells into the tissue, see Troubleshooting 5 to instantly respond to an imprecise cell injection.

13. Once all injections are finalized, cover the 6-well plate with the lid and incubate the plate with all brain slices for 4 h at 37°C, 5% CO_2_ in a humidified incubator.

### Monitoring and treatment of cells integrating into the mouse brain tissue

**Timing: Up to 10 days**

**Figure 7:**
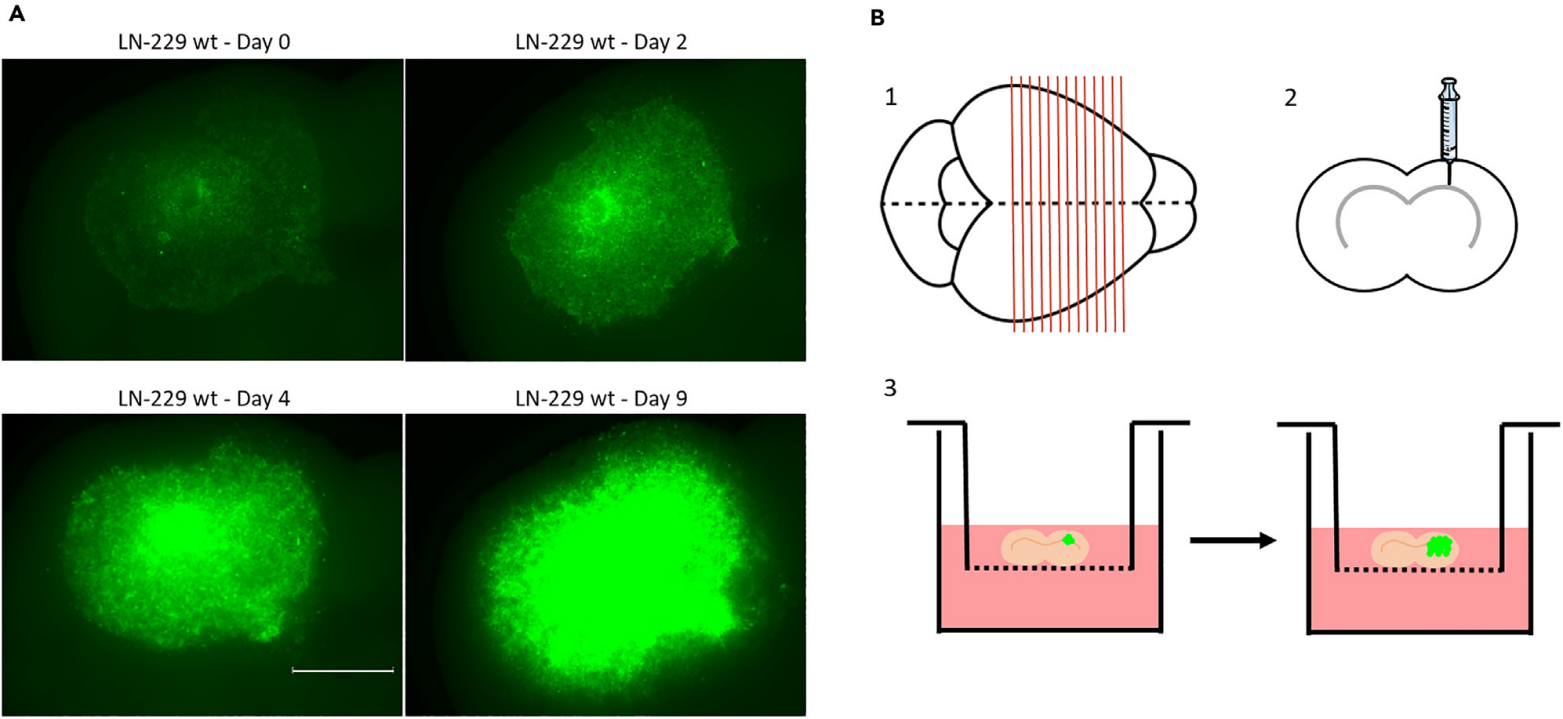
GFP positive cells integrated in brain slice (A) Representative images of brain slice after injection of GFP-positive GBM LN-229 wt cells during 9 days of culture. Increased GFP signal over time and GFP-positive area indicate continuous proliferation and invasion of tumor cells. Signal at Day 7 and Day 9 is already at the saturation level (scale bar: 1550 μm). Images were taken with an upright epifluorescent microscope. (B) Schematic overview of *ex vivo* mouse brain slice culture starting from 1; the process of cutting a brain into slices, 2; the injection of cells into the brain slice and 3; the invasion and thus colonization of injected cells into the mouse brain slice placed in a transwell chamber. Figure created with BioRender.com.

In this step of the protocol, integration and motility of injected tumor cells inside the mouse brain tissue will be monitored by fluorescence microscopy. Desired treatment(s) will be applied and mouse brain tissue slices will be maintained for up to ten days. A schematic overview shown in Figure 7B summarizes the up to this point executed steps.

14. After 4 h of incubation, verify the brain slices again at a fluorescent microscope. These images will represent the state of the brain slices before adding any treatment to their media (t = 0).

***Note:*** Capture one image per brain slice. Image the slice in the respective fluorescence channel, as well as in the bright-field mode. Choose one magnification, set the focus accordingly and record these settings for further imaging.

**CRITICAL:** If you observe an increased background fluorescence in the brain slices, presumably originating from remaining blood, perfusion of the brain to remove circulating blood in the blood vessels prior to the mouse sacrifice is recommended, see Troubleshooting 6.

15. Administer desired treatment.
  ***Note:*** Chemical treatments can be directly applied in the culture medium or directly into the tumor.
  a. Pre-warm the required volume of brain slice medium, supplement it with the desired treatment.
  b. Fill the wells of the culture plate harboring the brain slices with 1 mL of the provided medium.
16. Determine empirically the start of your treatment, considering the growth and invasion characteristics of your tumor cell lines.

***Note:*** Depending on the type of study, you may consider administering the treatment of choice at later time points. In our study, we administered the treatment to the brain slice medium four hours after the cell injection since we aimed to study the effect of the treatment on early stages of invasion.

17. 48 h post injection, remove the culture medium from below the brain slices by soft aspiration.
18. Directly fill the wells of the culture plate harboring the brain slices with 1 mL of fresh pre-warmed brain slice medium, including the required treatment(s) if needed.
19. Verify the brain slices under the fluorescent microscope and confirm the presence and invasion status of tumor cells. Record fluorescence and brightfield applying the same settings as on t = 0 (Figure 7A and Methods video S7). Record the respective time point, the number or label of the brain slice and the scale.
20. Repeat step 17–19 every 48 h to reassure that the injected cells are viable and your treatment(s) still potent. In case of a reduction in tumor cell number or viability, see Troubleshooting 7.
21. Continue the experiment for maximum of seven to ten days at 37°C. Determination of the experiment’s endpoint should be empirically tested taking into account the invasive capacity of the injected cells and the administered treatment and/or conditions.

***Note:*** Out of the ten brain slices we used to inject tumor cells per experimental condition, generally we were able to finally analyze eight slices. On average 20% of the brain slices had to be excluded from the final analysis, which was mainly due to problems during cell injection into the brain slice.

**CRITICAL:** More detailed explained, the release of cells from the pipette tip during injection ending up below or on top of the tissue and starting to grow on the outer cell layer of the tissue slice instead of inside the brain tissue will exclude the slices from any further analysis. Tissue slices wherein the tissue became necrotic during the experiment need to be excluded from such as well.

22. At the endpoint of the experiment, in the respective case Day 10 of the brain slice incubation, image the brain slices in the bright-field and green fluorescent channel (488 nm) taking pictures every 2 h, ideally for the time course of 24 h.

***Note:*** These images of the brain slices taken every two hours will be required to analyze the velocity of the cells in step 32. Your image set will then consist of 12 pictures per brain slice.

***Note:*** We imaged the brain slices for later analysis using the IncuCyte microscope. However, we believe that the repeated imaging every two hours is also possible with another microscope. Using the IncuCyte live imaging system comes along with the advantage of CO_2_ (5%) and temperature (37°C) being controlled throughout the experiment. In this case, one can leave the brain slices in the machine during imaging.

***Note:*** If using another microscope, one may have to transport the well plate containing the brain slices in between the standard incubator and the microscope. Thus it may be difficult and laborious in finding back the area (and the focus) of interest to always track the same cell. Acquisition was done using the 4x objective.

***Optional:*** If possible, live cell imaging should be planned during the experimental procedure to evaluate single cell velocity. We performed live imaging for 24 h at the end point of the experiment (Day 10 of the brain slice incubation). Settings included imaging in the bright-field and the green channel (488 nm), using the 4x objective with pictures taken every two hours.

***Note:*** Saturation of the fluorescent signal of the colonized area should be avoided by adjusting the appropriate incubation time of the brain slices after cell injection. The incubation of such might be stopped shortly before reaching any saturation in the fluorescent signal.

***Note:*** Nonetheless, for later analysis of the colonized area, the intensity of the fluorescent signal will not be taken into account. In our case, rather the entire green fluorescent area was measured, independently of any saturation. Saturation of the fluorescent signal at the endpoint may rather be taken as a mean to assess and confirm the length in days for the incubation of the brain slices.

Methods video S7. Imaging of the area of colonization of GPF positive cells in the mouse brain slice tissue using the IncuCyte live cell imaging system Movie is centered on the side of colonization, related to step 19.

### Quantification and statistical analysis

Here we present two possible strategies to quantify tumor growth as well as migration and invasion characteristics of tumor cells inside mouse brain tissue. The first analysis aims at the measurement of the entire area of tumor cell colonization taking into account all cells that were injected into the mouse brain (Methods video S7). The second quantification focuses on tracking single cells and thus comprises the measurement of individual cell velocity (Methods videos S8, S9, S10, and S11). In our case, ImageJ software was used for image processing.

Methods video S8. Tracking of cell movement over time of a non-invasive cell line using IncuCyte Live cell imaging system Related to analysis of individual cell velocity tracking, step 48

Methods video S9. Tracking of cell movement over time of an invasive cell line using IncuCyte Live cell imaging system Related to analysis of individual cell velocity tracking, step 48

Methods video S10. Tracking of cell movement over time of a highly invasive cell line using IncuCyte Live cell imaging system Related to analysis of individual cell velocity tracking, step 48

Methods video S11. Live cell imaging of cell movement with two differently labeled cell populations (GFP, RFP) using IncuCyte Live cell imaging system Related to analysis of individual cell velocity tracking, step 48

#### Area of colonization

**Figure 8:**
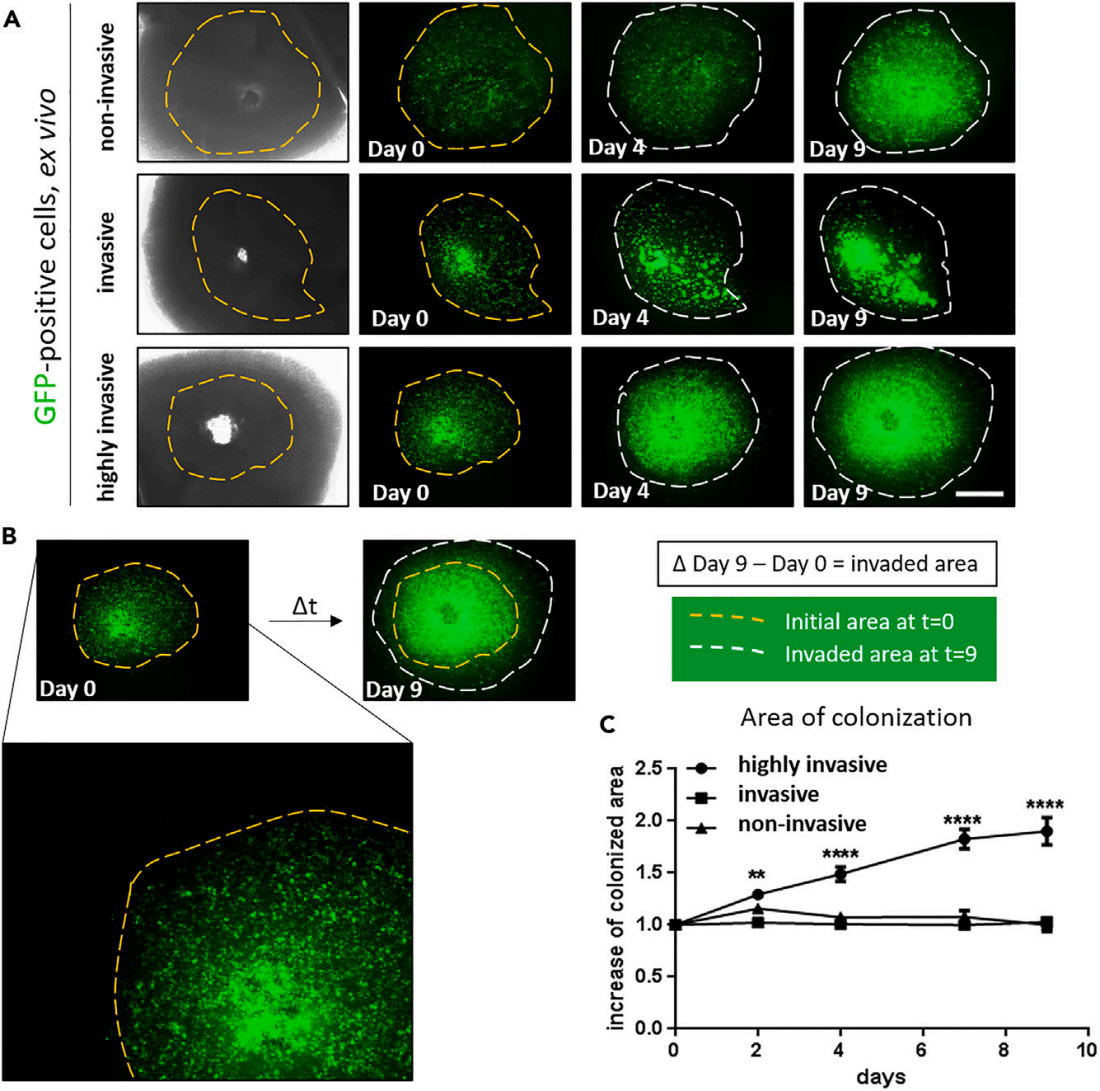
Representative pictures of GBM cells in *ex vivo* brain slices with their area of colonization (A) The area of colonization of fluorescently labeled GBM cells is shown over time (9 days after injection): Three different cell lines were analyzed and classified into non-invasive, invasive and highly invasive cells based on the measured area of colonization (scale bar 1000 μm). (B) The area of colonization (outlined with dotted white lines in the representative images) was determined using ImageJ and is represented in the graph. Images in A and B were taken with an upright epifluorescent microscope. (C) Quantification of increase of colonized area of GBM stem-like cells in *ex vivo* brain slice cultures 9 days after tumor implantation (n = 10), displayed as average ± SEM. Modified from Schuster et al., Nat. Com, 2018.^2^

To assess the general area of colonization by tumor cells, measure the area of tumor growth over time. A comprehensive exemplary result of such analysis for invasion of GBM cells is shown in Figures 8A–8C. Consider a potential impact of tumor cell proliferation over time. By acquiring images of the brain slices every 48 h and measuring the colonized area in ImageJ, it has to be kept in mind that not only invading cells are analyzed, but also proliferating cells, see Troubleshooting 8.

**Figure 9:**
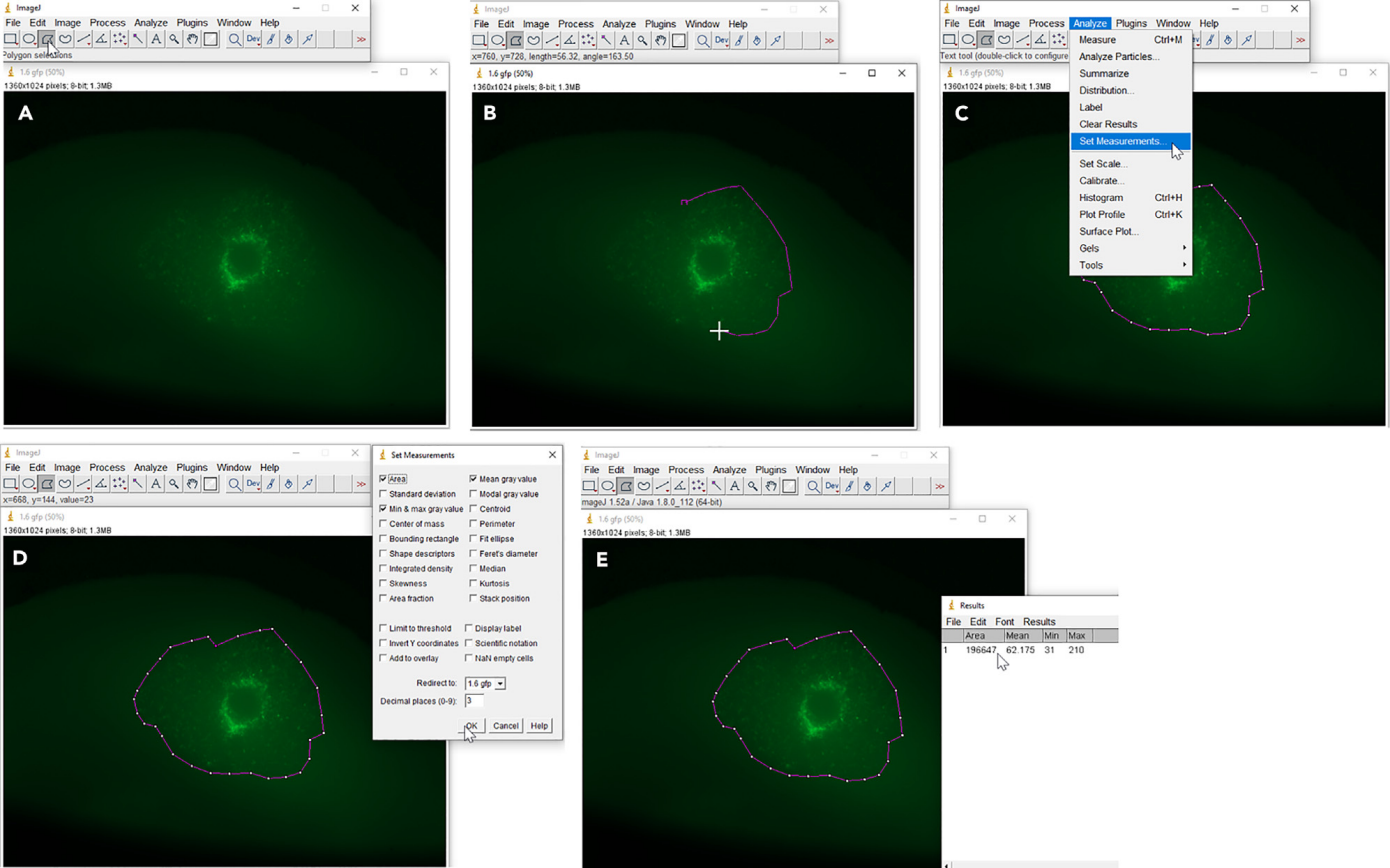
Steps of ImageJ analysis of exemplary brain slice microscopy images to measure the area of cell invasion (A) Selection of polygon tool to manually start edging the colonized area (B). (C and D) Two options are displayed to execute the measurement of the encircled area. (E) Results window shows the quantification of the measured area.

23. Transfer the images taken of the brain slices for all time points (these should include t = 0, t = 48 h, t = 96 h,…, t = endpoint) to one folder.
24. Open the image of t = 0 in the ImageJ software and select the polygon tool (Figure 9A).
25. Edge the colonized area, which is the region where the fluorescent cells accumulated within the tissue slice (Figure 9B).
26. Once completed, press CTRL+M to measure the encircled area (Figure 9C).

***Note:*** Alternatively, you can also navigate to “analyze”, then “set measurements” and thus select “area”, redirect it to the image to be analyzed and confirm by selecting “ok” (Figure 9D).

**Figure 10:**
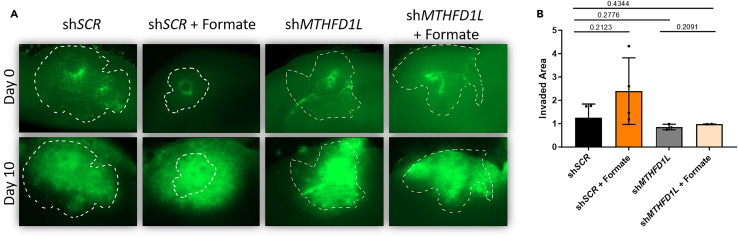
Brain slices imaged after injection of LN-229 sh*SCR* and LN-229 sh*MTHFD1L*^1^ cells and respective treatment with 500 μM of sodium formate (Day 0) and following 10 days of incubation (Day 10) (A) The area of green fluorescent labeled cells was encircled (with white dotted lines) for every condition at Day 0 and the same border was overlaid for the images taken at Day 10. This enables to easily visualize the area of colonized cells throughout the incubation from Day 0 to Day 10. Images were taken with an upright epifluorescent microscope. (B) Representation of the area of colonized cells over time (numeric analysis). Dots represent biological replicates of independent experiments ± SEM.

27. Proceed with the later time point images one after the other. To do so, open the image of the next time point, and again edge manually the colonized area.
28. Once all images have been analyzed with the polygon encircled area, copy the quantification of the measured area being displayed in the results window (Figure 9E) and paste the values in an Excel file.
29. Normalize all values to time point 0 (t = 0) as ratio, by calculating the fold change between t = 0 and any subsequent measurement.
30. Once you analyzed all images, select CTRL+W to close the windows in ImageJ.
31. You can depict the data as a barplot (Figure 10B) and by representative pictures of the edged colonized area (Figure 10A).

#### Tumor cell velocity

Assessing the velocity of a single cell enables detailed investigation of the process of migration and invasion summarized as the motility at the single cell level without a possible bias linked to the proliferation of the cell(s). Tumor cell velocity can be assessed with ImageJ, analyzing at least 15 randomly selected single cells per condition and ten technical replicates per cell line (LN-229 sh*SCR*, LN-229 sh*MTHFD1L*^1^). For random selection of the single cells, consider them not touching the border region of the brain slice, their level of fluorescence being sufficient for subsequent microscopy analysis and the selected cells remaining in the field of view during the course of the analysis. Take images accordingly and save these in an ImageJ compatible format (8-bit).

32. Export the respective image set from the IncuCyte software, as explained in the specific instrument manual (https://www.sartorius.com/en/products/live-cell-imaging-analysis/live-cell-analysis-software).

***Note:*** Based on the IncuCyte scan pattern set to two hours, our image set consisted of 12 pictures per brain slice.

**Figure 11:**
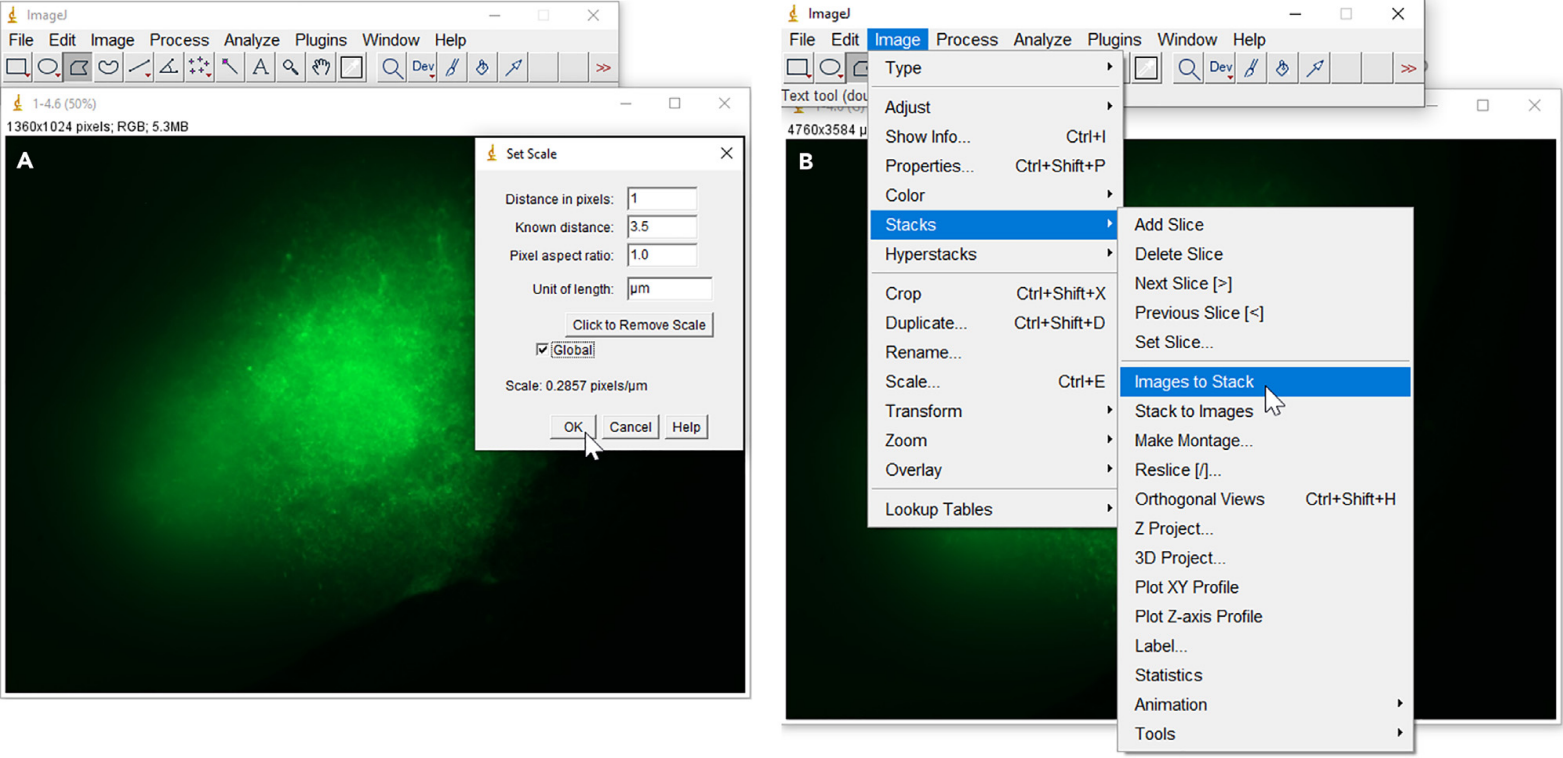
First steps including parameter settings for analysis of cell velocity in ImageJ ImageJ settings for (A)“Set scale” and (B)“Images to Stack”.

33. To determine the velocity of the cells, the ImageJ software plugin MTrackJ, an ImageJ Plugin for Motion Tracking and Analysis, is required and can be freely downloaded from https://imagescience.org/meijering/software/mtrackj/.
34. Download the plugin “MTrackJ” and install it using the command Ctrl+Shift+M in ImageJ.
35. Once completed, open the IncuCyte software and select relevant images.
36. Start with one experimental condition and select the first brain slice.
37. Once completed, select “image set” and introduce an ID for the images to be exported.
38. Export the images in a .tiff file and subsequently open them in the ImageJ software.
39. Select “analyze”, then “set scale” and another window will pop up.
40. Set the parameters for “the distance in pixels” to “1”, the “known distance” to “3.5” and the “unit of length” to “μm” (Figure 11A). Concluding, 1 pixel corresponds to a distance of 3.5 μm, a scale that will be applied to any future measurements in this type of analysis.

***Note:*** Theses dimension parameters (in pixels) depend on the IncuCyte instrument version (or on the respective instruments if other microscopes are used for this type of analysis). In every situation, you will have to perform a setting up calibration in ImageJ first, that converts the initial pixel measurement into meaningful measuring units.

41. Tick the box “global” and confirm the settings by selecting “ok” (Figure 11A).
42. Navigate to “images”, scroll to “stacks” and select “images to stack” (Figure 11B). In another window, you can name your stack.

***Note:*** Other live-cell imaging setups may be used as well to perform the described analysis. A major requirement is the recording of an identical part of the brain slice during 24 h, so that you are able to monitor the same cells for every time point that you are going to analyze.

**Figure 12:**
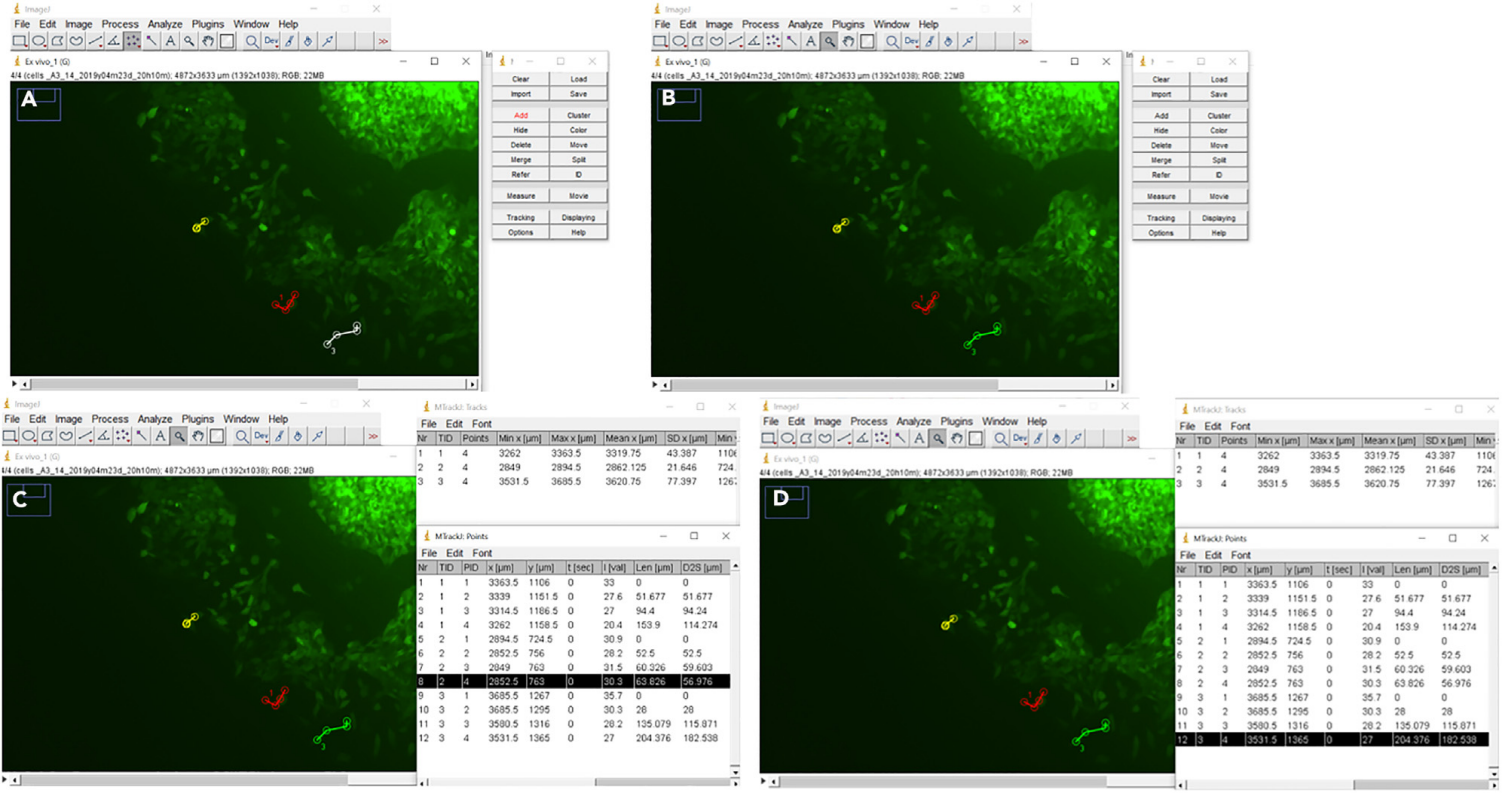
Overview of MTrackJ step by step analysis in ImageJ (A and B) Tracking of single cells in image stack. Moving distance measured for (C) cell n°2 highlighted in yellow and (D) cell n° 3 highlighted in green.

43. To measure the distance the cells moved over time, open the image stack and navigate to “Plugins” and therein select “MTrackJ”.
44. Select “Add” and thus the button will light up in red meaning it is active to actually select cells (Figure 12A).
45. After having clicked centrally on a randomly selected cell, the image for the following time point will automatically appear where you then need to click on the same cell selected on the first image.
46. Repeat step 45 for all images from the stack (Figure 12A).
47. Once finalized, confirm your selection by clicking “Add” again and the button will change color from red to inactive black (Figure 12B).
48. Repeat step 44–47 for as many cells as you want to analyze.

***Note:*** In our case, we analyzed 15 cells per brain slice (Methods videos S5 and S6).

***Note:*** Ensure to always start with the first image of the stack for the analysis of the single cell velocity in order to start at the earliest time point of the cell’s movement tracking.

49. After selecting all cells, select “Measure” and two further windows will open, MTrackJ: “Tracks” and MTrackJ: “Points” (Figure 12C).

***Note:*** The “Tracks” window displays an overview of all individual cells that were selected for analysis. The other window, “Points”, provides an overview of the respective selected cell (TID) in all images (PID) of the stack. Hereof, the values determined in μm for “x”, “y” and “len” (final length) are needed for further analysis (Figures 12C and 12D).

***Note:*** In order to graphically represent the **displacement of the cells in a bar plot**, values in column “Len [μm]” are needed. These values describe the distance one cell moved over time.

**Figure 13:**
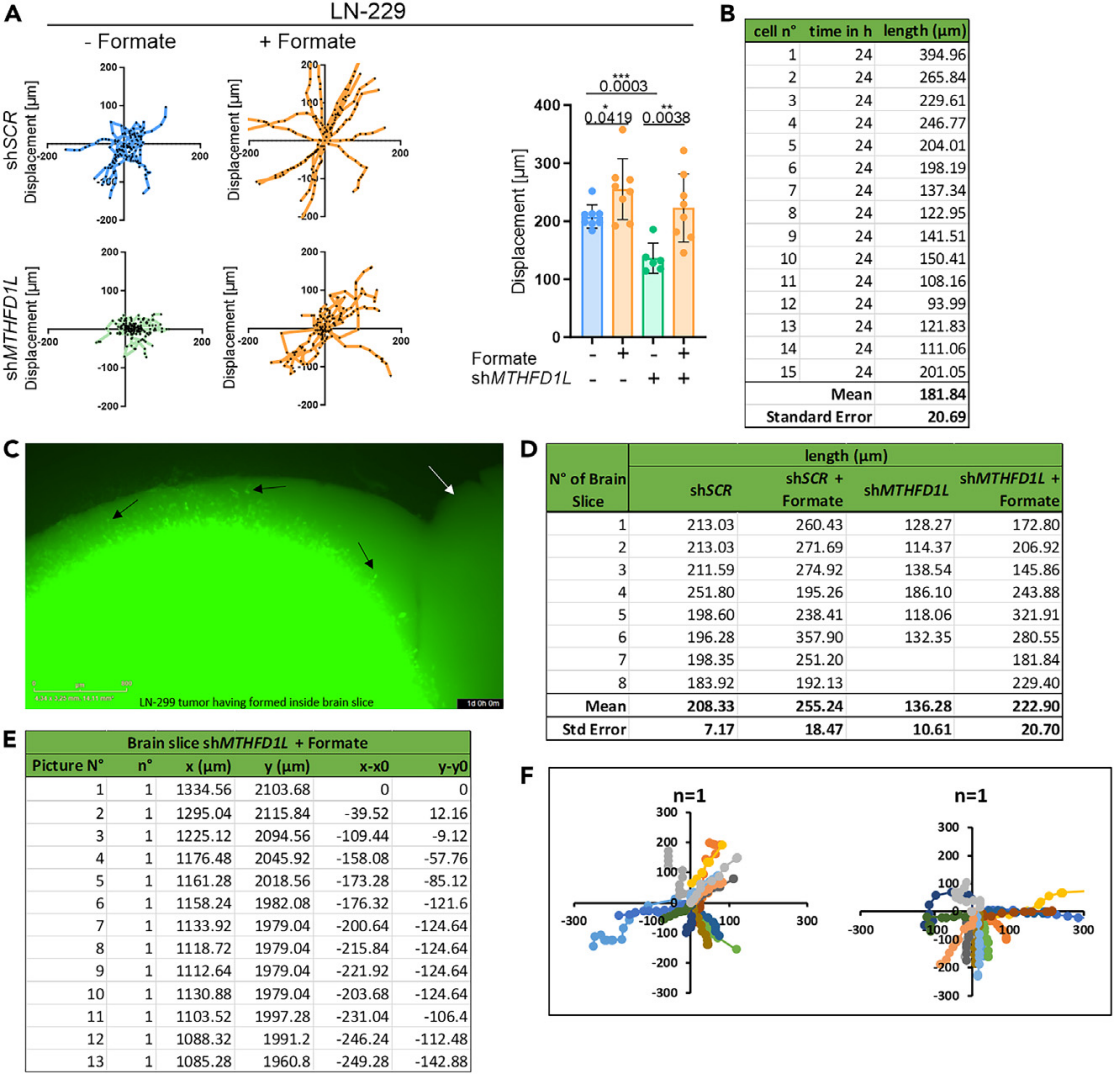
Assessment of velocity of injected cells inside the mouse brain slice (A) Displacement (in μm) (distance that cells moved) of LN-229 cells in regard to sh*SCR* vs. MTHFD1L KD cells +/− 500 μM of sodium formate. Each colored line shown in the scatter plot corresponds to the movement/displacement of one cell, with each dot showing the cell at the respective time point (every 2 h). Dots in the barplot represent mean displacement of 15 cells, n = 6–8, mean ± SEM; unpaired t-test with Welch’s correction. Modified from Delbrouck et al., 2023, Cell Reports.^1^ (B) Average length (in μm) of 15 cells per brain slice and their arithmetic mean with standard deviation. (C) Exemplary microscopy image showing bright green fluorescent LN-299 cells (exemplarily indicated by black arrows) moving inside the brain tissue (light green structure, indicated by white arrow). Image taken with IncuCyte live cell imaging system, scale bar 800 μm. (D) Summary of the average length of monitored cells per brain slice over all experimental conditions. This table represents the raw data to depict the barplot in A. (E) Summary of coordinates “x[μm]” and “y[μm]” for one single monitored cell in brain slice n°1 over 13 analyzed pictures. Travelled distance per image shown as values for x-x_*0*_ and y-y_*0*_. (F) Displacement of monitored cells in a scatter plot. Each scatter plot shows the displacement of cells (individually colored in plot) in one brain slice (*n* = 1).

50. Copy the “Len [μm]” value at the end point of your live cell imaging (in our case it was 24 h, see step 22) into an excel file. These values summarize the cell’s velocity.
51. Repeat step 50 for every single cell that you have selected in this brain slice.
52. In excel, calculate the average (the arithmetic mean) of the values for the length measured for each cell at time point 24 h.
53. In the same excel file, open another sheet and repeat step 35–52 for every imaged brain slice within that single experimental condition.
54. Open another sheet in the same excel file, copy paste all recently gained arithmetic mean values for the average length of 15 cells per brain slice and calculate their arithmetic mean (Figure 13B). Repeat this step for all brain slices in all tested experimental conditions (Figure 13D).

**CRITICAL:** If you plan to show the displacement of the cells in a scatter plot, do not close the MTrackJ: “Points” window. You will need to copy paste certain values from this window in step 58.

55. In addition to the arithmetic mean, also calculate the standard error by dividing the standard deviation of all values by the square root of the number of samples.
56. Finally, you can depict the mean moved distance (displacement of the cells) within one experimental condition, e.g., LN-299 sh*MTHFD1L*^1^ (Figure 13A, right bar plot, green bar).
57. Repeat steps 35–56 to compare the displacement of the cells in all experimental conditions.

***Note:*** In order to graphically represent the **displacement of the cells in a scatter plot**, values in column “x[μm]” and “y[μm]” are needed. These values describe the location of the individual cell in the respective image.

***Note:*** In our experiment, we visualized the displacement of 15 analyzed LN-299 *shMTHFD1L*^1^ cells per single brain slice, averaging eight individually analyzed brain slices (n = 8, except *shMTHFD1L* n = 6) as shown in Figure 13A on the left in green. Figure 13C represents exemplary microscopy image showing bright green fluorescent LN-299 cells (indicated by black arrows) moving inside the brain tissue.

58. Open another excel file and therein an additional sheet.
59. Copy paste the values resulting from the tracking of one single cell in the image from “x[μm]” and “y[μm]” into two separate columns (Figure 13E).
60. Annotate both coordinates x and y for the starting point from which the selected cell was shown to move as “0”.
61. Individually subtract any later x and y coordinate respectively of this selected cell from the value at “0”, so the starting position of the analyzed cell.
62. Repeat step 59–61 for every single cell monitored in the respective image set (brain slice).
63. You will end up with as many *x-x*_*0*_ and *y-y*_*0*_ values, as you analyzed pictures for each cell in the individual brain slice.
64. Use the values *x-x*_*0*_ and *y-y*_*0*_ resulting from every single cell tracking in the respective brain slice as data source to generate a scatter plot (Figure 13F).

## Expected outcomes

The protocol allows for the assessment and quantification of cancer cell invasion in viable mouse brain slices. Monitoring of colonization of the injected tumor cells allows to characterize the process of cancer cell tumor growth and invasion in a more physiological environment compared to simple *in vitro* conditions. One rather straightforward readout of the assay focuses on the assessment of the area of colonized tumor cells. Since here the invasion area may be impacted by the proliferation rate of the tumor cells, the assessment of velocity of the single cells brings additional insights into the behavior of tumor cells in the brain. Importantly, the protocol allows to assess tumor growth and motility capacities of different cell cultures, including upon genetic engineering and treatment over several days at lower cost than *in vivo* experiments.

## Limitations

The main limitation of the protocol is the need for a fluorescent labeling readout allowing to track viable tumor cells inside the mouse brain tissue structure. Injecting tumor cells without a fluorescent marker excludes follow-up over time by live-cell imaging and requires additional immunohistochemistry-based readouts at fixed time points. While assessment of the area of colonization can be performed with a basic fluorescence microscope, tracking single cells requires additional equipment allowing for live fluorescence imaging. Discrimination between tumor cell proliferation and invasion is challenging in certain tumor cell models, thus advanced single cell tracking may be required to distinguish between the two processes. Another main limitation of the protocol is the fact that with the described analysis, one cannot fully exclude that the cells being analyzed in the microscopy images and/or videos may be migrating on top or even underneath the tissue. We would here suggest a technique that will allow for acquisition of z stacks of the brain slices, to confirm the cells’ integration into the mouse brain tissue. An additional limitation is linked to the finite viability of brain slices beyond 10–12 days in culture, which restricts protocols requiring longer treatment exposure times. To further assess the brain tumor structure and crosstalk with normal brain cells additional steps may involve paraffin-based fixation of the tissue followed by immunohistochemistry^4^ or spatial omics-based assessment. Optionally, specific genetically engineered mice with fluorescently labeled cell populations may be applied as alternative. The co-cultures can also involve depletion and/or integration of other cell types e.g., immune cells that may integrate to brain slices over time.^5^ Advanced cancer models such as tumor tissue fragments and organoids may be more difficult to integrate to brain slices *ex vivo*. Alternatively, *ex vivo* cultures can be obtained from brains with already developed xenografted tumor tissue. Since brain microenvironment is significantly altered in patient-derived orthotopic xenografts,^6^^,^^7^ additional optimization of tissue cutting may be needed.

## Troubleshooting

### Problem 1

During the cutting process, the mouse brain tissue slices start attaching to the razor blade instead of quickly removing from the blade after being cut to remain as a whole cut brain together on the filter paper.

### Potential solution

Immediately stop the cutting process to release the slice from the blade. To do so, take the tweezers and carefully remove the slice from the blade. If you manage to remove without inducing any damage to the tissue, you can still keep this slice for injection. Carefully transfer it into the Petri dish filled with cutting solution to fully submerge it with liquid. However, if the tissue appears already damaged or with injured tissue structures, discard the respective slice. Clean off the blade from any remaining tissue using 70% EtOH and tissue paper. Verify if the brain is still in the correct position to the blade, by confirming the blade’s perpendicular position to the brain. Flip the reset switch to continue with the cutting process.

### Problem 2

You are facing difficulties during cutting due to the stickiness of the brain. After having stopped the cutting process within the cutting of a mouse brain, it may be difficult to restart the entire process, due to the stickiness of the tissue and the cut tissue slices.

### Potential solution

To prevent difficulties to restart the process, rather focus on working with fresh mouse tissue. If you have access to additional mouse brains, proceed directly with a freshly isolated and ice-cold brain. Discard the part of the leftover brain that you will not use anymore. Keep in mind that the more time passes during the cutting of one mouse brain, the more the temperature inside the tissue increases. Such temperature increase will result in the tissue becoming stickier. Cutting of ice-cold brain tissue will be of advantage here, since the process will have to be stopped less times due to sticking of slices to the blade. If you do not have access to additional mouse brain material, you need to make a compromise in trying to use as much of the brain as you can. Make sure that the slices that you keep for further experimentation are free of any tissue damage and disrupted tissue parts.

### Problem 3

You are facing difficulties during the disassembling of the single tissue slices from the cut brain and the slices do not detach when removing the single filter paper stripes.

### Potential solution

Depending on personal preferences, use the tweezers, the flat spatula or two brushes and carefully separate the cut slices from each other (Figures 5B and 5C). When using tweezers, detach brain slices one by one from the whole brain using the tweezers (preferred option). When using the spatula or the two brushes, carefully “peel off” the slices from the brain. Use some magnifying glasses to ensure precise movements. It may also be helpful to use one pair of tweezers to fix the filter paper and the other pair or the flat spatula to separate the brain slices. Be extremely careful not to damage the thin tissue slices. Only keep and further process the slices that still maintain all essential structures. Ensure that the tissue slice is still intact as a whole.

### Problem 4

The injection of an exact amount of cells in a rather small volume of 1 μL in ECM is challenging since you need to be sure to precisely release all cell solution from the pipette tip.

### Potential solution

To ensure that the same cell number is injected, the cells of the remaining aliquot of all injections can be counted to see if the content of cells was correct. Furthermore, once having injected the 1 μL suspension containing the cells, verify that the pipette tip does not retain any liquid. If still the case, you may try other pipetting methods such as reverse pipetting to release the volume of 1 μL precisely.

### Problem 5

It may be difficult to precisely inject all cells resuspended in the very small volume of 1 μL into the brain slice tissue, without encountering cells being released below or on top of the tissue. This step is very crucial to the entire protocol, since it will limit the risk of encountering false results due to cells growing on top or below the tissue slice resulting in incomparable replicates between different experimental conditions. Moreover, partial integration of cells into the brain tissue needs to be prevented.

### Potential solution

To ensure that the cells are appropriately injected into the tissue and do not end up on top or below the tissue slice, ensure that once the pipette tip has punched a hole in the tissue, at the same time you release the cell suspension from the pipette tip and simultaneously you also carefully but immediately remove the pipette tip horizontally from the tissue. In case of ending up with a brain slice where cells were not completely injected into the brain slice tissue, try to remove those cells from above or below the brain with a pipette tip. You may also try to carefully flush the tissue slice with brain slice medium in order to rinse off the cells on or below the tissue. This can be considered as a washing step. Fully aspirate this medium afterwards. It may be advised to always ensure complete tissue integrity being a crucial prerequisite for correct injection of the cells. Another very helpful verification step before cell injection comprises assessment of the physical depth of the tissue slice. To do so, completely penetrate the tissue with an empty pipette tip to get a feeling of the actual thickness of the tissue. This will allow you to improve cell injection into the tissue slice.

### Problem 6

Brain tissue generally presents high autofluorescence, which can be further augmented by the blood retained in the blood vessels. Thus, if fluorescent tagging of tumor cells is low or the baseline autofluorescence of the cells is rather high, it may be difficult to precisely define the area of colonization and track single cells to determine the cells’ velocity. The increased background may introduce an error in the downstream image analysis and needs to be reduced.

### Potential solution

Instead of directly euthanizing the mice by cervical dislocation, first perfuse the heart of the mice using at least 10 mL of ice-cold 1x PBS. Perfusion with PBS removes blood from the blood vessel in the brain, which may decrease the overall autofluorescence of the brain tissue. Importantly, only authorized personal is allowed to perform perfusion and this procedure requires terminal anesthesia. Thereafter you can isolate the brain and pursue with the cutting. This step of perfusion reduces the risk for encountering high background during fluorescence microscopy. Another alternative of reducing the background fluorescence in the brain slice may be to prolong the incubation time of the freshly cut tissue, currently set to 18–24 h (step p), in order to allow more time for passive diffusion of the blood from the tissue into the surrounding medium. Since in our setting, the incubation time of 18–24 h worked well with the cell line we used and the subsequent analysis, we did not further investigate longer incubation times in the scope of this protocol. Optionally, you can apply fixation of the brain slice tissue^4^ or even enhance quality of the fluorescence microscopy images by clearance of the tissue as described by Eisemann et al., 2018.^4^

### Problem 7

You are observing that the injected cells and/or your freshly isolated brain slices at the time or even at a later stage after injection of the cells do not retain their expected viability or morphology. This means that your brain slices and the injected cells do not tolerate the above described brain slice medium.

### Potential solution

Test survival of tumor cells in the brain slice medium prior injection into the brain slices. You can test the survival of the tumor cells in the respective culture medium by mean of proliferation assays. Alternatively, you can also use the modified brain slice medium, a 1:1 mixture of Hibernate A-medium (Thermo Fisher Scientific, Cat. No. A1247501), 20% BIT-100 (Provitro, Cat. no. 2043100), 100 U/mL of Pen/Strep and DMEM-F12 (Thermo Fisher Scientific, Cat. no. 11320033), 20% BIT-100, 100 U/mL of Pen/Strep, 200 mM L-glutamine (Thermo Fisher Scientific, Cat. no. 25030081), 1 U/mL of heparin (Sigma, Cat.no. H3393). Store the medium in a dedicated closed container at +4°C to ensure proper cooling of the solution until the start of the experiment. Use this medium to culture your mouse brain slices in.

### Problem 8

When measuring the area of colonization by means of the increase in fluorescent signal, you cannot exclusively discriminate between invasion and proliferation. You need to assess the proliferation characteristics of the respective cells to more precisely conclude on their invasion characteristics.

### Potential solution

In order to have a mean for the proliferative capacities of the cells you inject into the mouse brain slices, you can, in parallel, perform proliferation assays. Retain the same experimental conditions for the cells as for the brain slices. This will give you an idea of the growth characteristics of the cells you inject into the brain slices and may hint to some *in vitro* growth differences between your tested conditions.

## Resource availability

### Lead contact

Further information and requests for resources and reagents should be directed to and will be fulfilled by the lead contact, Johannes Meiser (johannes.meiser@lih.lu).

### Technical contact

Technical questions on executing this protocol should be directed to and will be answered by the technical contact, Laura Neises (laura.neises@lih.lu).

### Materials availability

This study did not generate new unique reagents.

### Data and code availability

Original/source data for figures in the paper is available via https://doi.org/10.1016/j.celrep.2023.113034 and https://doi.org/10.1038/s41467-020-20029-y.

## Acknowledgments

We thank the LIH animal facility for providing the isolated mouse brains and reviewing the manuscript. We acknowledge the financial support by the Luxembourg Institute of Health, the Luxembourg National Research Fund (FNR) (ATTRACT program A18/BM/11809970, C21/BM/15718879/1cFlex, C20/BM/14646004/GLASSLUX, PRIDE15/10675146/CANBIO, and PRIDE19/14254520/i2TRON), Fondation Cancer (INVGBM), and the Televie-FNRS (ImmoGBM 7.8505.20, ImmoGBM2 7.6603.22). For the purpose of open access, and in fulfilment of the obligations arising from the FNR grant agreement, the author has applied a Creative Commons Attribution 4.0 International (CC BY 4.0) license to any Author Accepted Manuscript version arising from this submission. Figures were created with BioRender.com.

## Author contributions

Conceptualization, J.M. and L.N.; methodology, L.N., C.D., A.S., M.R., K.E., C.F., A.O., S.P.N., A.G., and J.M.; software, L.N., C.D., and A.S.; validation, L.N., C.D., M.R., K.E., and J.M.; formal analysis, L.N., C.D., C.F., M.R., K.E., and J.M.; data curation, C.D., A.S., C.F., M.R., A.G., and J.M.; writing – original draft, L.N., A.G., and J.M.; writing – review and editing, all authors; visualization, C.D., A.S., M.R., L.N., and J.M.; supervision, S.P.N., A.G., and J.M.; project administration, S.P.N., A.G., and J.M.; funding acquisition, S.P.N., A.G., and J.M.

## Declaration of interests

The authors declare no competing interests.
